# Butyrate combined with niacin enhances intestinal barrier function repair in weaned piglets infected with ETEC by promoting colonic metabolism and antimicrobial peptide expression

**DOI:** 10.1186/s40104-026-01405-y

**Published:** 2026-05-13

**Authors:** Qingsong Tang, Rui Zhen, Bijing Yang, Zhenyan Miao, Yangyang Wei, Shihui Ruan, Yiyi He, Yunxia Xiong, Qiwen Wu, Li Wang, Zongyong Jiang, Hongbo Yi

**Affiliations:** 1 State Key Laboratory of Swine and Poultry Breeding Industry, Key Laboratory of Animal Nutrition and Feed Science in South China, Ministry of Agriculture and Rural Affairs, Guangdong Provincial Key Laboratory of Animal Breeding and Nutrition, Institute of Animal Science, Guangdong Academy of Agricultural Sciences, Guangzhou, 510642 China; 2 Zhaoqing Branch Center of Guangdong Laboratory for Lingnan Modern Agricultural Science and Technology, Zhaoqing, 526238 China; 3 Heyuan Ruichang Feed Co., Ltd., Heyuan, 517000 China

**Keywords:** Butyrate, Colonic barrier function, Enterotoxigenic *Escherichia coli*, Niacin, Weaned piglets

## Abstract

**Background:**

Weaning piglets are highly susceptible to enterotoxigenic *Escherichia coli* (ETEC) infections, which can cause intestinal barrier function dysfunction and death. However, there is still a lack of efficient, economical, and safe nutritional interventions. This study aimed to investigate the effects of combining butyrate with niacin on intestinal barrier function repair and resistance to ETEC infection in weaned piglets. In this study, two 14-d animal experiments were designed to observe the optimal butyrate-to-niacin ratio and assess their responses to the ETEC challenge.

**Results:**

Supplementation with butyrate and niacin at a ratio of 100:2 (2,000 mg/kg butyrate and 40 mg/kg niacin, BN2) increased the average daily gain (ADG) and reduced the diarrhea incidence. We also observed an increase in the levels of nicotinamide adenine dinucleotide (NAD) in the colon of weaned piglets. Notably, BN2 promoted amino acid anabolism in the colon and enhanced glycolysis and the tricarboxylic acid (TCA) cycle by increasing the acetylation of key enzymes in the TCA. Furthermore, BN2 enhanced the expression of indispensable genes for the colonic mucosal barrier, including antimicrobial peptides such as porcine β defensin 1 (*pBD1*), porcine β defensin 2 (*pBD2*), and proline-arginine rich 39-amino acid peptide (*PR39*), tight junction proteins, and improved colonic microbiome composition. Based on these findings, we found that BN2 alleviated growth restriction and diarrhea, and modulated the expression of antimicrobial peptides, tight junction proteins, and cytokines to reduce colonic barrier function dysfunction in weaned piglets challenged with ETEC. Mechanistically, we confirmed that BN2 elevated the protein expression of acetylation of histone 3 lysin 27 (H3K27ac) and enhanced the binding of acH3K27 to the promoter regions of *pBD1* and *PR39*.

**Conclusions:**

Supplementation with BN2 improved growth performance, supported colonic barrier function repair, and enhanced disease resistance in weaned piglets challenged with ETEC. This offers new insights into nutritional strategies for intestinal barrier function repair of piglets infected with ETEC.

**Supplementary Information:**

The online version contains supplementary material available at 10.1186/s40104-026-01405-y.

## Background

Early weaning (at 3–4 weeks of age) exposes piglets to nutritional and environmental stress, which compromises the physiological function, morphology, immunity, and microbial balance of the intestine [1, 2]. As such, weaned piglets are highly susceptible to pathogenic bacteria, which increases pathogen colonization in the colon, particularly enterotoxigenic *Escherichia coli* (ETEC) [3]. ETEC produces adhesion factors and enterotoxins (heat-stable and heat-labile enterotoxins), which subsequently increase the secretion of colonic fluids and electrolytes, leading to diarrhea and growth retardation [4]. Additionally, ETEC infections alter intestinal metabolism and immune responses, exacerbating intestinal dysfunction [5]. ETEC infections are widespread globally, especially in developing countries, causing considerable losses to the livestock industry. Currently, the pig industry is still actively seeking effective nutritional interventions to mitigate ETEC-induced colonic barrier dysfunction.

Intestinal metabolism and barrier function are bidirectionally, dynamically, and deeply coupled. The interaction between energy metabolism and the intestinal barrier is crucial for maintaining intestinal physiological homeostasis. Damage to the intestinal barrier may lead to metabolic dysregulation, such as alterations in the gut microbiota, which impede the production of short-chain fatty acids (SCFAs) [6]. SCFAs serve as crucial substrates for intestinal energy metabolism. Butyrate, a key component of SCFAs in the intestines, is recognized for its central role as an energy source. Butyrate generates nicotinamide adenine dinucleotide (NADH) within mitochondria through the tricarboxylic acid cycle and β-oxidation, thereby promoting energy metabolism processes such as oxidative phosphorylation. Simultaneously, intestinal energy metabolism serves not only as an energy source but also as an intrinsic driver and regulator of maintaining intestinal barrier homeostasis. This primarily provides the energy for intestinal injury repair and upholds the integrity of the intestinal barrier, thereby reducing the risk of intestinal infection by ETEC [7]. Recent studies confirm that the gut's own metabolic activities do not passively receive protection from the barrier, but actively participate in constructing and maintaining barrier function [8].

Niacin, also known as vitamin B_3_, is a precursor of NAD and an essential nutrient [9]. Studies have showed that niacin influences various biological processes, such as transcription, mitochondrial biogenesis, the cell cycle, and apoptosis, by increasing histone deacetylation via NAD-dependent sirtuins [10, 11]. Thus, niacin may promote the butyrate-derived energy supply in the intestines by synthesizing NAD, and regulate gene expression of intestinal barrier function by modulating histone deacetylation. However, butyrate interacts with G protein-coupled receptors and inhibits histone deacetylases, potentially promoting gene expression by increasing histone acetylation [12]. Therefore, butyrate and niacin may exert antagonistic effects on regulating histone acetylation, making it crucial to clarify their dose–response relationships. However, the impact of varying ratios of butyrate and nicotinic acid on histone acetylation levels and the mechanisms underlying their regulation of antimicrobial peptide expression remain unclear. Furthermore, studies investigating the combined regulation of histone acetylation by butyrate and nicotinic acid to resist ETEC infection are still lacking.

Thus, the primary objective of this study was to investigate the effects of butyrate combined with niacin on colonic metabolism and resistance to ETEC-induced colonic barrier dysfunction in weaned piglets. Additionally, the mechanism by which butyrate combined with niacin regulates colonic metabolism and barrier function was elucidated through the analysis of protein acetylation modifications.

## Materials and methods

### Coating of butyrate and niacin

A dual-coating technique was used to coat sodium butyrate and niacin at the following ratios: 100:1, 100:2, and 100:8. The coat sodium butyrate and niacin at the ratios of 100:1, 100:2, and 100:8 contained the same dose of sodium butyrate (2,000 mg/kg), and niacin was supplemented with 20, 40, 80, and 160 mg/kg, respectively. Briefly, a carrier mixture (including hydroxypropyl methylcellulose, chitosan, silicon dioxide, etc.) is first swelled at high temperature, and then, sodium butyrate and niacin, dissolved in water at the correct proportions, are sprayed onto the surface of the carrier mixture. The carrier mixture is dried and slowly cooled to form solid particles. Finally, a coating material is applied to the surface of the sodium butyrate‒niacin acid mixture particles. The main components of the coating material are Brazilian carnauba wax, palm fat and polyethylene glycol.

### Experimental procedure and sample collection

#### Experiment 1

A total of 96 weaned piglets (Duroc × Landrace × Yorkshire, average age of 21 d) with an initial body weight (BW) of 5.68 ± 0.10 kg were randomly allocated to four dietary treatments. Each treatment consisted of six replicate pens, and there were four weaned pigs per pen, balanced for sex. The pigs in the control (CON) group were fed a basal diet. The pigs in the butyrate and niacin at ratios of 100:1 (BN1) group, 100:2 (BN2) group, and 100:8 (BN8) group were fed basal diet supplemented with a coating of butyrate and niacin at ratios of 100:1, 100:2, and 100:8, respectively. The nutritional composition of the basal diets met the requirements recommended by the National Research Council (2012) [13] and is showed in Table 1. Piglets were housed in enclosed barns with slatted floors, and the room temperature is maintained at 25–30 °C. The experimental diets were provided at 8:00, 12:00, 16:00 and 20:00 daily, and all the piglets had ad libitum access to water and diets. The pigs were fed for 14 d. On d 15, one piglet per replicate was randomly selected to be anesthetized by an anterior vena cava injection of sodium pentobarbital (40 mg/kg BW) and then sacrificed. Carefully incise the abdominal cavity of the pigs. Approximately 200 mg of contents from the middle colon were collected and preserved in a 2-mL sterile cryovial, in triplicate. After longitudinal sectioning to expose the lumen of the middle colon, irrigate with pre-chilled saline. Collect approximately 200 mg of middle colonic tissue and store in a 2-mL cryovial. Prepare nine identical copies, then snap-frozen in liquid nitrogen, and stored in the refrigerator at −80 °C for measurement. In addition, middle colonic segments with complete cross-sections were collected, placed in 4% paraformaldehyde, and stored at room temperature for measurement. These samples underwent analysis for NAD levels, metabolomics, acetylation omics, enzyme activity, qRT-PCR, Western blot, microbial composition, SCFA concentrations, hematoxylin and eosin (HE) staining, and immunohistochemistry.

**Table 1:** Composition and nutrient level of basal diet (as-fed basis), %

Ingredient	Content, %	Nutrient level^2^	Content, %
Corn starch	48.50	Digestible energy, kcal/kg	3,792
Low protein whey powder	15.00	Crude protein	20.09
Soybean protein	15.50	SID lysine	1.44
Whey protein concentrate	8.00	SID methionine + cysteine	1.01
Inulin	3.00	SID threonine	0.82
Soybean oil	1.00	SID tryptophan	0.27
Saccharose	3.00	Ca	0.80
Choline chloride (50%)	0.30	STTD P	0.41
NaCl	0.35		
NaHCO_3_	0.15		
KCl	0.50		
MgSO_4_	0.30		
CaHPO_4_	1.50		
L-Lysine	0.25		
DL-Methionine	0.15		
L-Threonine	0.10		
Premix^1^	1.50		
Total	100.00		

^1^The premix provided following per kg of the diet: vitamin A, 11,000 IU; vitamin B_12_, 87.5 μg; vitamin B_1_, 5 mg; vitamin B_2_, 17.5 mg; vitamin B_6_, 10 mg; biotin, 0.25 mg; vitamin D_3_, 1,100 IU; vitamin K, 2.5 mg; vitamin E, 80 IU; D-calcium pantothenate, 50 mg; folic acid, 1.5 mg; Zn (ZnSO_4_·H_2_O), 289 mg; Fe (ferrous fumarate); 333 mg; Cu (CuSO_4_·5H_2_O), 24 mg; Mn (MnSO_4_·H_2_O), 12 mg; and Se (Na_2_SeO_3_), 0.69 mg; I (CaI_2_O_6_), 0.23 mg

^2^Except for measured values of the crude protein and calcium, others are calculated values

#### Experiment 2

A total of 24 weaned piglets (Duroc × Landrace × Yorkshire, average age of 21 d) with an initial BW of 6.61 ± 0.23 kg were randomly allocated to four treatments. Each treatment consisted of six replicate pens, and there were four weaned pigs per pen, balanced for sex. The pigs in the CON group and ETEC K88 infection (K88) group were fed a basal diet, while the pigs in the BN2 group and ETEC K88 infection with BN2 dietary treatment (KB2) group were fed a BN2 diet. Pigs in the K88 and KB2 groups were orally administered 10 mL of ETEC K88 bacterial solution at a concentration of 4 × 10^9^ CFU/mL on d 3, 7, and 11 of the experiment. Pigs in the CON and BN2 groups were orally administered equal volumes of saline. The basal diet was the same as that used in Exp. 1 (Table 1). Piglets were kept at the same temperature, feeding schedule and management program as those used in Exp. 1. The pigs were fed for 14 d. On d 15, six piglets per group were anesthetized via an anterior vena cava injection of sodium pentobarbital (40 mg/kg BW), then sacrificed. Carefully incise the abdominal cavity of the pig and collect approximately 200 mg of liver tissue and 200 mg of spleen tissue, placing each into separate 2‑mL cryovials. After longitudinal sectioning to expose the lumen of the middle colon, irrigate the lumen with pre-chilled saline. Collect approximately 200 mg of middle colonic tissue and store in a 2-mL cryovial, in duplicate. Snap-frozen in liquid nitrogen, and then stored at −80 °C for measurement. In addition, middle colonic segments with complete cross-sections were collected, placed in 4% paraformaldehyde, and stored at room temperature for measurement. These samples underwent analysis for HE staining, qRT-PCR, Western blot, immunohistochemistry, and immunofluorescence.

### Growth performance and diarrhea incidence

To evaluate the growth performance of the pigs, the weaned piglets were weighed on d 0, 7 and 14 of the Exp. 1, and the piglets were weighed on d 0 and d 14 of the Exp. 2. The average daily gain (ADG), average daily feed intake (ADFI), ratio of gain to feed (G/F) were calculated at the end of the Exp. 1 and Exp. 2. The diarrhea index and diarrhea incidence were calculated with reference to our previous study [14]. In brief, diarrhea scores follow a three-point scale, with scores of 2 or higher indicating diarrhea. Since no piglets in the Exp. 1 exceeded a diarrhea score of 2 throughout the entire experimental period, this study did not analyze the diarrhea incidence for Exp. 1.

### NAD and NADH level assay

The levels of NAD and NADH in the colonic tissue of piglets from Exp. 1 were measured using the NAD/NADH Quantitation Kit (Sigma, USA). Each biological replicate measured 1 sample, with 6 samples measured per group. Briefly, approximately 20 mg of colonic tissue is added to 400 μL of NADH/NAD buffer. After homogenization, the mixture is centrifuged at 12,000 × *g* for 5 min. Transfer the supernatant to a 10 kDa MWCO ultrafiltration tube (Merck, Billerica, MA, USA) and centrifuge at 13,000 × *g* for 15 min. The extract in the collector is Solution A. Half of Solution A is treated in a 60 °C water bath for 30 min to yield Solution B. Add the following reaction mixture to the microplate: 30 μL of Solution A or Solution B, 98 μL of NAD Cycling Buffer, and 2 μL of NAD Cycling Enzyme Mix. After incubating at room temperature for 5 min, add 10 μL of NADH Developer and incubate for 1–4 h. After terminating the reaction, measure the absorbance at 450 nm. The protein concentration in the sample was determined using the BCA Protein Assay Kit (Beyotime, Shanghai, China). The results calibrated using protein concentration.

### Metabolomic analysis

Metabolomic analysis of the colonic contents of piglets from Exp. 1 were performed using the ultraperformance liquid chromatography‒tandem mass spectrometry (HPLC‒MS/MS). Each biological replicate measured 1 sample, with 6 samples measured per group. An AB Triple TOF 5600/660 mass spectrometer (AB SCIEX) coupled with an Agilent 1290 Infinity LC ultrahigh-pressure liquid chromatograph was used for the analysis. Approximately 60 mg of colonic contents were homogenized in 200 μL of saline. After vortexing for 60 s, 800 μL of a methanol/acetonitrile (v:v = 1:1) solution was added, and the mixture was shaken well. The samples were sonicated at low temperature for 30 min twice, then placed at −20 °C for 1 h and centrifuged at 14,000 × *g* for 20 min. Then, the supernatant was collected for LC‒MS analysis. After the original data were converted, the differentially abundant metabolites were screened on the basis of a variable importance plot (VIP) > 1 and *P* < 0.05, and KEGG enrichment analysis was subsequently performed on those metabolites.

### Acetylation omics analysis

Approximately 50 mg of colonic tissue per piglet from Exp. 1 was weighed, and four times the volume of the lysis buffer (8 mol/L urea, 1% protease inhibitor, 3 μmol/L trichostatin A, 50 mmol/L nicotinamide) was added before samples were subjected to ultrasonic lysis. The mixture was subsequently centrifuged at 12,000 × *g* for 10 min at 4 °C, after which the supernatant was collected. An equal amount of protein from each sample was used for enzymatic hydrolysis. The peptides were subsequently dissolved in liquid chromatography mobile phase A and separated via a NanoElute ultrahigh-performance liquid chromatography phase system (Bruker, Billerica, MA, USA). Mobile phase A was an aqueous solution containing 0.1% formic acid and 2% acetonitrile; mobile phase B was acetonitrile containing 0.1% (v/v) formic acid. The mobile phase gradient settings were as follows: 0‒44 min, 6%‒22% B; 44‒56 min, 22%‒35% B; 56‒58 min, 35%‒80% B; and 58‒60 min, 80% B. The flow rate was maintained at 450.00 nL/min. The peptides were separated via the UHPLC system, injected into the capillary ion source for ionization and then analyzed via an Orbitrap Fusion Tribrid mass spectrometer (Thermo Fisher Scientific, Waltham, MA, USA). The ion source voltage was set at 2.0 kV, and the peptide parent ions and their secondary fragments were detected and analyzed via high-resolution TOF. The secondary mass spectrometry data were retrieved via MaxQuant 1.6.6.0. The cysteine alkyl carbamidomethyl (C) was set as a fixed modification, and the variable modifications were ['Acetyl (Protein N-term)', 'Oxidation (M)', 'Acetyl (K)']. The quantification method was set to LFQ, and the FDRs for protein identification and PSM identification were set to 1%. Each biological replicate measured 1 sample, with 6 samples measured per group.

### Enzyme activity assay

Approximately 100 mg of colonic tissue per piglet from Exp. 1 was weighed, homogenized with the extraction solution from the specific kit, centrifuged, and the supernatant was collected for analysis. Each biological replicate measured 1 sample, with 6 samples measured per group. The activities of hexokinase (HK), pyruvate kinase (PK), succinate dehydrogenase (SDH), methanol dehydrogenase (MDH), and ATP-citrate lyase (ACLY) in the colon were determined via commercial kits. The kits for HK, PK, SDH, and MDH were obtained from Nanjing Jiancheng Bioengineering Institute (Nanjing, China), and the ACLY kit was obtained from Beijing Solarbio Science & Technology Co., Ltd. (Beijing, China). The protein concentration in the sample was determined using the BCA Protein Assay Kit (Beyotime, Shanghai, China). All procedures were performed according to the manufacturers’ instructions. The results calibrated using protein concentration.

### Histomorphological analysis

Briefly, paraformaldehyde-fixed colonic segments of piglets from Exp. 1 and Exp. 2 were dehydrated, embedded in paraffin, cut into 5-μm thick sections and stained with HE. Each biological replicate measured 1 sample, with 6 samples measured per group. The morphology was observed under a microscope (DM1000, Leica Microsystems, Buffalo Grove, IL, USA), and the length of the submucosa was measured.

### Quantitative real-time polymerase chain reaction (qRT-PCR)

The mRNA expression was measured according to the previous study [15]. In brief, total RNA was extracted from approximately 20 mg of colonic tissue of piglets from Exp. 1 and Exp. 2 using TRIzol (#15596026, Invitrogen, Carlsbad, CA, USA). Each biological replicate measured 1 sample, with 6 samples measured per group. The total RNA was quantified via a NanoDrop 1000 spectrophotometer (Thermo Fisher Scientific, Waltham, MA, USA). The cDNA was subsequently synthesized by reverse transcription of RNA via the PrimeScript RT reagent Kit (TaKaRa, Japan), and was then subjected to qPCR. The reaction mixture (10 μL) contained 5 μL of iTaq Universal SYBR Green Mix, 0.5 μL of forward primer (10 μmol/L), 0.5 μL of reverse primer (10 μmol/L), and 4 μL of 10 × diluted cDNA template. The relative mRNA level for each gene was calculated via the 2^−ΔΔCt^ method. The primer sequences are shown in Table S1.

### Western blot

Approximately 100 mg of colonic tissue per piglet from Exp. 1 and Exp. 2 was placed in 1 mL of radio immunoprecipitation assay lysis buffer (RIPA) containing a 1% protease inhibitor cocktail. Randomly select 3 biological replicates, with 3 samples measured per group. After mechanical homogenization, the mixture was placed on ice for 30 min to lyse, followed by centrifugation at 10,000 × *g* for 5 min. The protein concentration in the supernatant was determined using the BCA Protein Assay Kit (Beyotime, Shanghai, China). Equivalent amounts of protein were diluted to equal concentrations using 5 × loading buffer (Beyotime, Shanghai, China) and denatured at 100 °C for 5 min. Subsequently, the samples were separated by sodium dodecyl sulfate polyacrylamide gel electrophoresis (SDS‒PAGE), and the proteins were transferred to polyvinylidene fluoride (PVDF) membranes. The membranes were incubated overnight at 4 °C with primary antibodies and then incubated for 1 h at room temperature with the appropriate secondary antibody. The blots were detected via Clarity Western ECL Substrate (Bio-Rad, Hercules, CA, USA). The protein band intensity was measured with ImageJ software (National Institutes of Health, MD, USA). All antibody information is showed in Table S2.

### Immunohistochemistry

The expression levels of zona occludin-1 (ZO-1) and mucin-2 (MUC-2) in the colonic segments of piglets from Exp. 1 and Exp. 2 were measured using immunohistochemistry. Each biological replicate measured 1 sample, with 6 samples measured per group. In brief, 5-μm-thick sections were subjected to antigen retrieval, peroxidase elimination, blocking, and incubation with primary antibodies against ZO-1 and MUC-2 overnight at 4 °C, followed by incubation with secondary antibody and diaminobenzidine (BL732A, Biosharp, China). Finally, the samples were photographed via microscopy and analyzed via ImageJ (National Institutes of Health, MD, USA). All antibody information is showed in Table S2.

### Microbial composition analysis

Genomic DNA was extracted from approximately 100 mg colonic contents per piglet from Exp. 1 using the cetyl trimethylammonium bromide method, followed by agarose gel electrophoresis to determine DNA purity and concentration. Each biological replicate measured 1 sample, with 6 samples measured per group. The diluted genomic DNA was used as a template to amplify the V3–V4 regions of the 16S rRNA genes using the forward primer 515 F and the reverse primer 806R. The amplified product was detected by electrophoresis with equal volume mixed samples, and the target product was subsequently recovered. The PCR products were recovered and purified via the GeneJETTM Gel Extraction Kit (Thermo Fisher Scientific, Wilmington, USA). The validated libraries were sequenced using the NovaSeq 6000 platform provided by Novogene (Beijing, China).

### Determination of SCFA concentrations

The concentrations of SCFAs in the colonic contents of piglets from Exp. 1 were measured using gas chromatography–mass spectrometry on a TRACE 1310-ISQ system (Thermo Fisher Scientific, Wilmington, USA). Each biological replicate measured 1 sample, with 6 samples measured per group. Briefly, 50 mg of colonic contents were weighed, 50 μL of 15% phosphoric acid, 100 μL of 125 μg/mL internal standard, and 400 μL of ether were added, and the contents were mixed well. The supernatant was collected after centrifugation at 12,000 × *g* for 10 min at 4 °C, after which the concentration of SCFAs was determined.

### Immunofluorescence

The 5 μm-thick sections from colonic segments of piglets from Exp. 2 were dehydrated, antigen retrieved, and incubated with bovine serum proteins for 30 min, and dilutions of interleukin 1 beta (IL-1β), interleukin 10 (IL-10), and tumor necrosis factor-α (TNF-α) were added dropwise after removing the sealing solution and incubated overnight at 4 °C. The sections were placed in phosphate buffered saline and washed 3 times for 5 min each. The secondary antibody was then added dropwise and incubated for 1 h at room temperature. The sections were incubated with 4',6-Diamidino-2-phenylindole dihydrochloride (DAPI) for 10 min in the dark, followed by the addition of an autofluorescence quencher, rinsing for 10 min, washing three times, and then sealing with an autofluorescence quencher. Images were obtained via a Zeiss LSM710 laser confocal scanning microscope (Zeiss, Germany) and analyzed via ImageJ (National Institutes of Health, MD, USA). Each biological replicate measured 1 sample, with 6 samples measured per group. All antibody information is showed in Table S2.

### Chromatin immunoprecipitation (ChIP)-qPCR

To analyze the binding of the porcine β defensin 1 (*pBD1*) and proline-arginine rich 39-amino acid peptide (*PR39*) promoters to acetylation of histone 3 lysine 27 (H3K27ac), the promoter region information of *PBD1* and *PR39* was initially analyzed via a web browser (http://segtp.jxau.edu.cn/pencode/?genome=susScr11). The binding of *pBD1* and *PR39* promoters to H3K27ac in the colonic tissue of piglets from Exp. 2 was detected using the SimpleChIP^®^ Plus Sonication Chromatin IP Kit (Cell Signaling Technology, Danvers, MA, USA). The 100 mg colonic tissue from the Exp. 2 were ground in liquid nitrogen and subjected to crosslinking in 1% formaldehyde for 10 min, followed by quenching with glycine. After 3 washes with PBS with protease inhibitor cocktail, the pellets were collected by centrifugation and resuspended in ChIP Sonication cell Lysis Buffer. Then further sonication was performed according to the manufacturer’s recommendations. Subsequently, anti-IgG/anti-H3K27ac antibodies and magnetic beads were added for co-incubation. The immunoprecipitate was then incubated with protein G magnetic beads, and the antibody–protein G magnetic bead complex was collected for elution, followed by DNA purification. Finally, purified ChIP DNA was collected for further ChIP-qPCR assays. ChIP-qPCR assays were performed according to the manufacturer’s recommendations. The anti-H3K27ac antibody are showed in Table S2. The primers of *pBD1* and *PR39* promoters are showed in Table S3.

### Statistical analysis

The statistical analyses were performed via SPSS22.0 statistical software (SPSS Inc., USA) and GraphPad Prism 8.0 (GraphPad Inc., USA). Data obtained during the study were normalized using Shapiro–Wilk normality test. Data conforming to a normal distribution were analyzed for differences using one-way analysis of variance (ANOVA) and Tukey's or Duncan's multiple comparisons. Data analysis of metabolomics and microbiomics was performed using the Kruskal-Wallis test. All the data are expressed as the mean ± standard error of the mean (SEM).

## Results

### Experiment 1: growth performance and colonic metabolism of weaned piglets

**Fig. 1:**
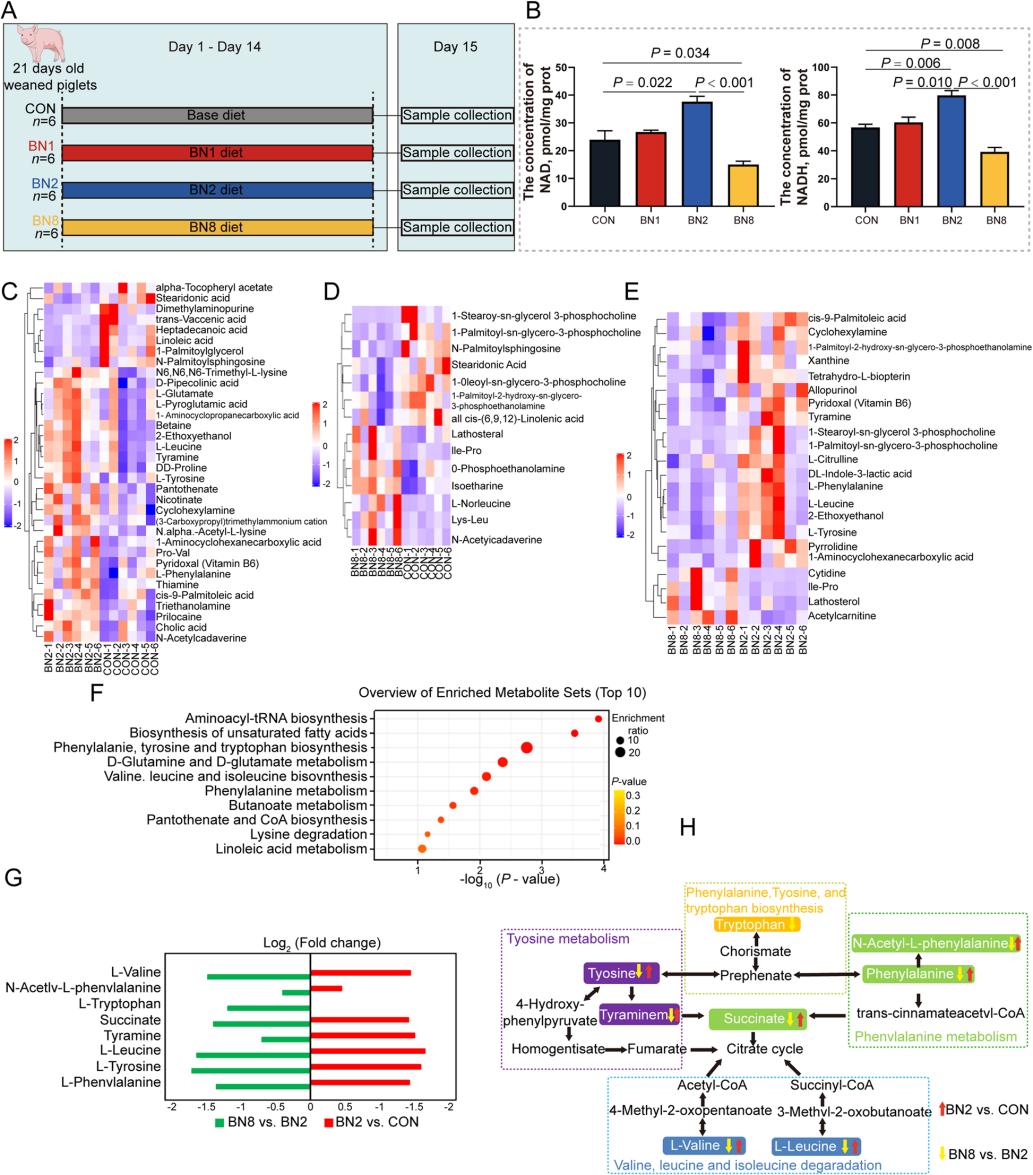
Butyrate combined with niacin promotes growth and colonic amino acid metabolism in weaned piglets. **A** Experimental design for Exp 1. **B** The concentrations of NAD and NADH in the colon. **C–E** Heatmap analysis of the changes in the metabolic profile in the colonic contents of BN2 vs. CON (**C**), BN8 vs. CON (**D**), and BN8 vs. BN2 (**E**). **F** Pathway analysis of differentially abundant metabolites between the BN2 group and the CON group. **G** Primary differential metabolites in the following pathways: phenylalanine metabolism; valine, leucine and isoleucine degradation; and the tyrosine metabolism pathway. **H** Schematic diagram of metabolic dynamics in the colon. Data are presented as mean ± SEM (*n* = 6)

First, a 14-d animal experiment was conducted to evaluate the effects of dietary supplementation with butyrate to niacin ratios of 100:1, 100:2, and 100:8 on the growth and colonic metabolism of weaned piglets (Fig. 1A). From d 1 to 14, the ADG of pigs was increased in the BN2 group compared with that of pigs in the CON and BN8 groups (*P* < 0.05, Table 2). The G/F of pigs was increased in the BN2 group compared with that of pigs in the CON, BN1, and BN8 groups from d 1 to 14 (*P* < 0.05). In addition, we found that the diarrhea index of pigs from d 1 to 14 of the experiment was higher in the BN8 group than in the CON, BN1, and BN2 group (*P* < 0.05). Notably, the concentration of NAD in the colon of the BN2 group was higher than that in the CON group and BN8 group (*P* < 0.05, Fig. 1B). Moreover, the concentration of NADH in the colon of the BN2 group was greater than those in the CON, BN1, and BN8 groups (*P* < 0.05). However, we observed that the concentrations of NAD and NADH in the colon of the BN8 group were lower than those in the CON group (*P* < 0.05). To further explore the effects of butyrate combined with niacin on colonic metabolism in piglets, we performed HPLC‒MS/MS analysis of colonic metabolites. We found that the BN2 group primarily increased the contents of amino acids and vitamins in the colon, such as L-glutamine, L-leucine, D-proline, L-tyrosine, L-phenylalanine, Pro-Val, N(alpha)-acetyl-L-lysin, pantothenate, nicotinate, thiamine, and pyridoxal (vitamin B_6_) (Fig. 1C). In contrast, the BN2 group decreased the content of fatty acids in the colon, such as alpha-tocopheryl acetate, stearidonic acid, heptadecanoic acid, and linoleic acid. Moreover, we observed an increase in the content of lathosterol, Ile-Pro, isoetharine, and N-acetylcadaverine in the colon and a decrease in the content of stearidonic acid and all *cis*-(6,9,12)-linolenic acid in the colon of the BN8 group compared with those in the CON group (Fig. 1D). The contents of amino acids and fatty acids in the colon, such as L-phenylalanine, L-leucine, L-tyrosine, Ile-Pro, *cis*-9-palmitoleic acid, 1-palmitoyl-2-hydroxy-sn-glycero-3-phosphoethanolamine, 1-stearoyl-sn-glycerol 3-phosphocholine, and 1-palmitoyl-sn-glycero-3-phosphocholine, were decreased in the BN8 group compared with the BN2 group (Fig. 1E). In addition, the BN8 group presented increased the contents of cytidine, Ile-Pro, lathosterol, and acetylcarnitine in the colon compared with the BN2 group. The metabolite enrichment pathways revealed that phenylalanine, tyrosine and tryptophan biosynthesis; D-glutamate and D-glutamate metabolism; valine, leucine and isoleucine biosynthesis; phenylalanine metabolism; and lysine degradation pathways were enriched in the BN2 group compared with those in the CON group (Fig. 1F). Among the metabolites related to the above metabolic pathways, the levels of L-valine, N-acetyl-L-phenylalanine, succinate, tyramine, L-leucine, L-tyrosine, and L-phenylalanine, were greater in the BN2 group than in the CON group, whereas the levels of these metabolites were lower in the BN8 group than in the BN2 group (Fig. 1G). Overall, the BN2 group promoted NAD synthesis and enhanced amino acid anabolism in the colon, with these amino acids potentially entering the citrate cycle (TCA cycle) to boost energy metabolism, whereas BN8 showed the opposite results (Fig. 1H).

**Table 2:** Effects of butyrate combined with niacin on growth performance and diarrhea index in weaned piglets

Items	CON	BN1	BN2	BN8	SEM	*P*-value
Day 1 BW, kg	5.617	5.592	5.650	5.750	0.1705	0.990
Day 7 BW, kg	8.325	8.743	9.117	8.731	0.2237	0.695
Day 14 BW, kg	11.22	11.36	12.35	11.14	0.309	0.505
Days 1 to 7						
ADG, g	386.9	450.2	495.2	425.8	16.33	0.115
ADFI, g	450.0	480.9	482.7	463.1	15.36	0.874
G/F	0.8651	0.9365	1.045	0.9181	0.02611	0.088
Days 7 to 14						
ADG, g	413.1	373.6	461.3	343.8	18.34	0.116
ADFI, g	519.6	548.8	569.6	459.5	18.53	0.170
G/F	0.7923	0.6823	0.8257	0.7435	0.02708	0.274
Days 1 to 14						
ADG, g	400.6^b^	412.4^ab^	478.9^a^	384.7^b^	13.45	0.040
ADFI, g	484.8	514.9	526.2	461.3	15.88	0.488
G/F	0.8288^b^	0.8033^b^	0.9193^a^	0.8320^b^	0.01424	0.012
Diarrhea index	0.2083^b^	0.2738^b^	0.2649^b^	0.9940^a^	0.09302	0.002

Mean and total SEM are listed in separate columns (*n* = 6)

^a,b^Different superscript letters within a row indicate significant differences (*P* < 0.05)

*BW* Body weight, *ADG* Average daily gain, *ADFI* Average daily feed intake, *G/F* Ratio of gain to feed

### Experiment 1: colonic protein acetylation modification of weaned piglets

**Fig. 2:**
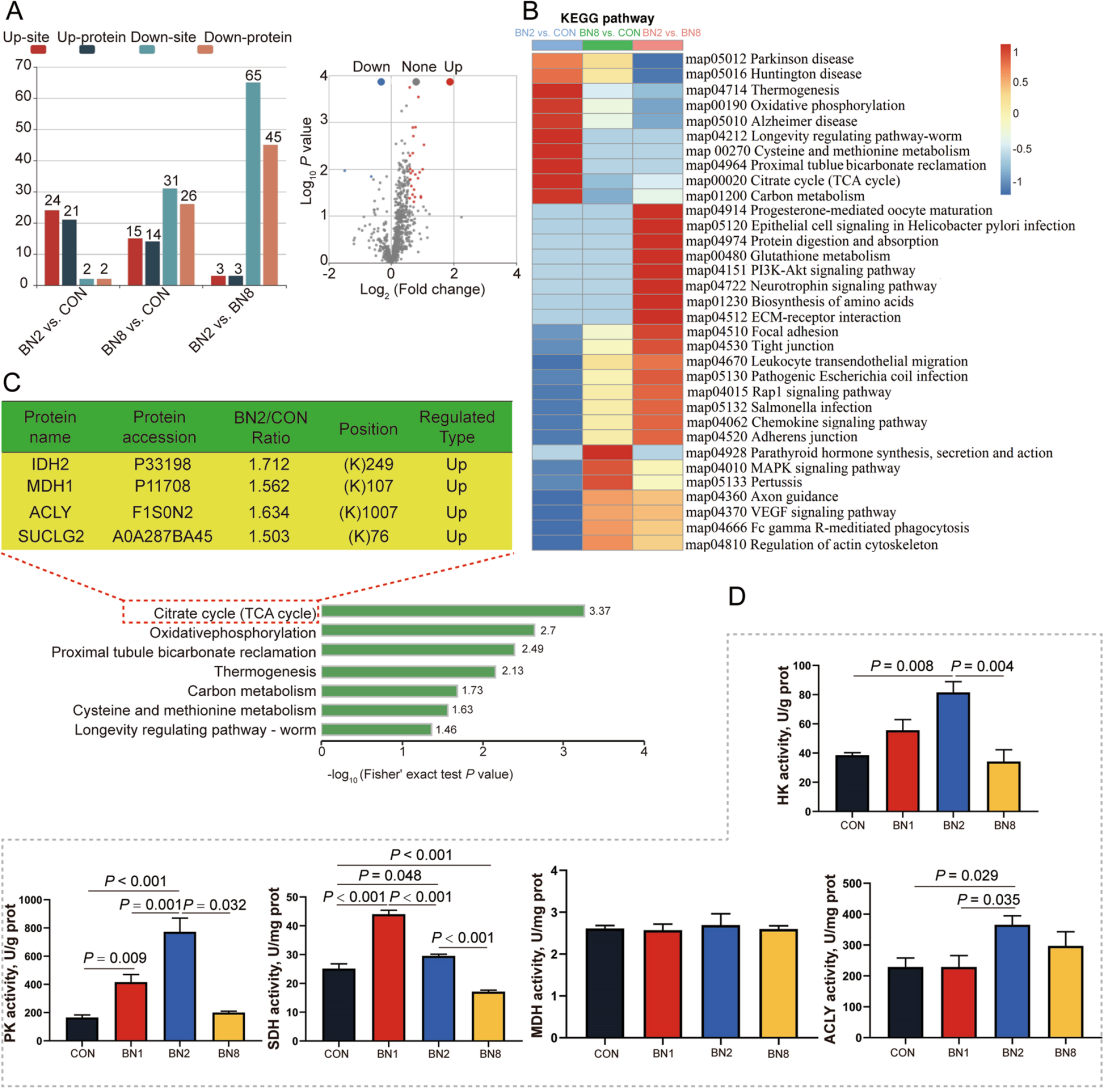
Butyrate combined with niacin promotes acetylation of key enzymes in the TCA cycle to enhance glucose catabolism. **A** The number of differentially modified proteins and modification sites in the colon. **B** KEGG enrichment analysis of differentially acetylated proteins. **C** Proteins acetylated of key enzymes in the TCA cycle. **D** The enzyme activities of glucose catabolism (HK, PK, SDH, MDH and ACLY) in the colon. Data are presented as mean ± SEM (data from acetylation omics, *n* = 3; data from enzyme activities, *n* = 6)

To further clarify the molecular mechanisms that alter colonic metabolism, we utilized a comprehensive quantitative acetylation proteomics approach to elucidate the specific effects of the combination of butyrate and niacin on protein acetylation patterns. All the differentially modified proteins and sites identified in this study are showed in Fig. 2A. Compared with the CON group, the BN2 group presented 21 upregulated proteins and 2 downregulated proteins (24 increased differential protein modification sites and 2 decreased differential protein modification sites), whereas the BN8 group presented 14 upregulated proteins and 26 downregulated proteins (15 increased differential protein modification sites and 31 decreased differential protein modification sites). In addition, the BN2 group presented 3 upregulated proteins and 45 downregulated proteins (3 increased differential protein modification sites and 65 decreased differential protein modification sites) compared with the BN8 group. As showed in Fig. 2B, compared with the CON group, the differentially modified proteins in the BN2 group were enriched mainly in energy metabolism pathways, including the TCA cycle, oxidative phosphorylation, cysteine and methionine metabolism, and carbon metabolism. Compared with the BN8 group, the differentially modified proteins in the BN2 group were enriched mainly in biosynthesis of amino acids, glutathione metabolism, tight junctions, and pathogenic *Escherichia coli* infection. Importantly, the BN2 group mainly changed the acetylation of key proteins in the TCA cycle, including isocitrate dehydrogenase 2 (IDH2), malate dehydrogenase 1 (MDH1), ACLY and succinate-CoA ligase GDP-forming subunit β (SUCLG2) (Fig. 2C).

To further explore the effects of the combination of butyrate and niacin on the glucose metabolic pathway (including the TCA cycle), we examined the activities of glucose metabolism-related enzymes. We found that the enzyme activities of PK and SDH in the colon were greater in the BN1 group compared with those in the CON group (*P* < 0.05, Fig. 2D). The enzyme activities of HK, PK, SDH and ACLY in the colon were higher than in the BN2 group compared with the CON group (*P* < 0.05). However, we found that the enzyme activity of SDH in the colon was lower than that in the BN8 group compared with those in the CON and BN2 groups, and the enzyme activities of HK and PK in the colon were lower than those in the BN8 group compared with those in the BN2 group (*P* < 0.05).

### Experiment 1: colonic antimicrobial peptide expression and intestinal barrier function of weaned piglets

**Fig. 3:**
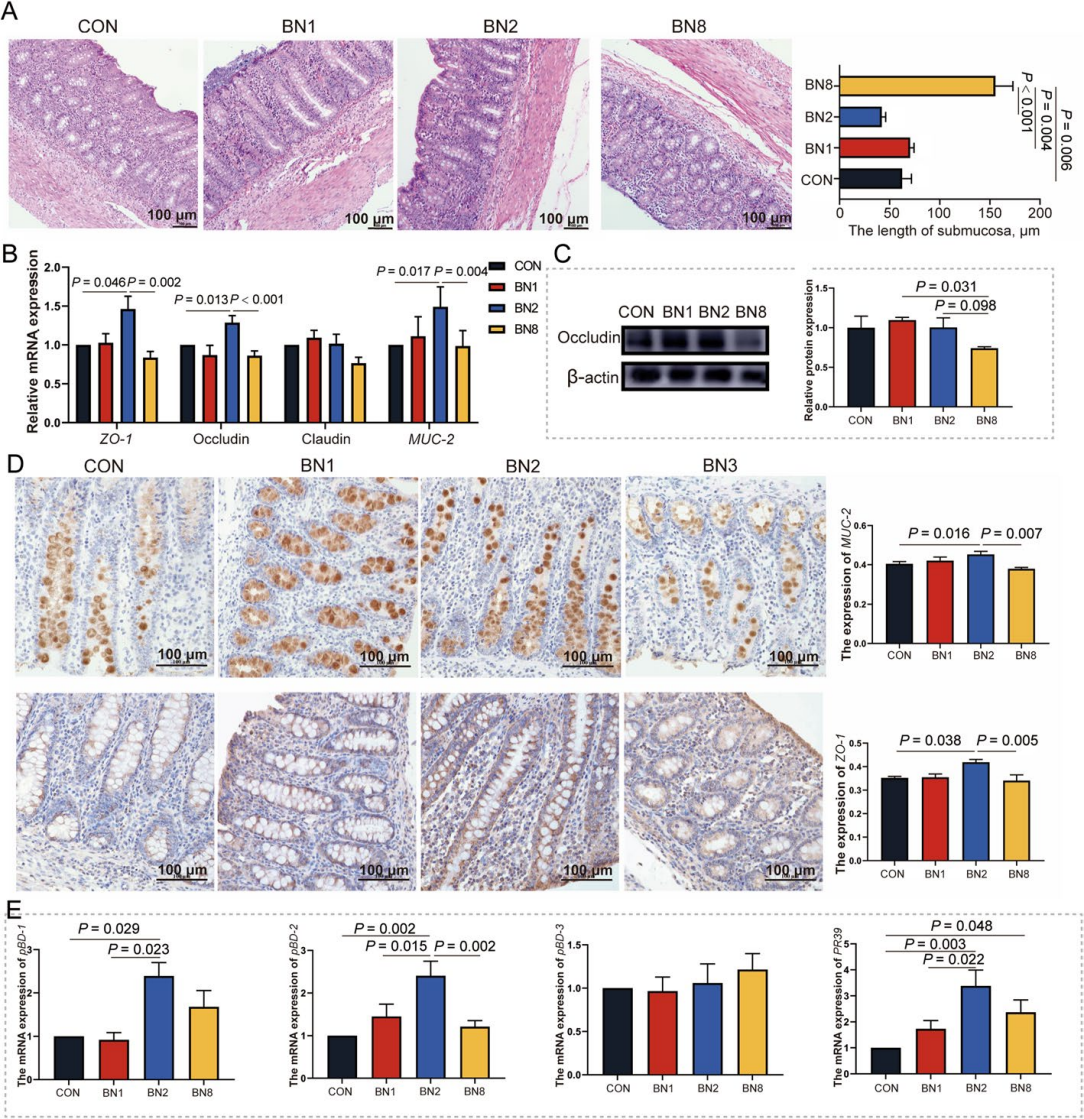
Butyrate combined with niacin enhances colonic antimicrobial peptide expression to improve intestinal barrier function. **A** The submucosal length in the colon was measured via HE staining (scale bar = 100 µm). **B** The mRNA expression of tight junction proteins (*ZO-1*, claudin-1, and occludin) and *MUC-2* in the colon. **C** The protein bands and quantitative analysis of occludin in the colon. **D** Immunohistochemical images and quantitative analysis of MUC-2 and ZO-1 in the colon. **E** The mRNA expression of antimicrobial peptides (*pBD1*, *pBD2*, *pBD3*, and *PR39*) in the colon. Data are presented as mean ± SEM (data from Western bolt *n* = 3; other data, *n* = 6)

To evaluate the regulatory effect of butyrate combined with niacin on colonic barrier function, we first examined colonic morphology of weaned piglets. We observed that the submucosal length in the colon of BN1, BN2, and BN8 groups was lower than those in the CON group (*P* < 0.05, Fig. 3A). Given the essential role of tight junction proteins and mucins in maintaining intestinal epithelial barrier integrity, we measured the mRNA expression of these proteins in colonic tissues. Notably, the mRNA levels of *ZO-1*, occludin, and *MUC-2* in the colon of the BN2 group were greater than those in the CON and BN8 groups (*P* < 0.05, Fig. 3B). However, the protein expression of occludin did not differ between the BN2 group and the CON group (*P* > 0.05, Fig. 3C). We also observed that the protein expression of occludin in the BN8 group was lower than those in the BN1 group (*P* = 0.031), and showed a tendency to increase in the BN8 group (*P* = 0.098). Further analysis by immunohistochemistry confirmed that the protein expression of ZO-1 and MUC-2 in the colon was higher than that in the BN2 group compared with that in the CON and BN8 groups (*P* < 0.05, Fig. 3D). Notably, the mRNA levels of *pBD1* and *PR39* in the colon in the BN2 group were greater than those in the CON and BN1 groups (*P* < 0.05, Fig. 3E). Moreover, the β defensin 2 (*pBD2*) mRNA level in the colon was greater in the BN2 group than in the CON, BN1, and BN8 groups (*P* < 0.05).

### Experiment 1: colonic microbial composition and SCFAs

**Fig. 4:**
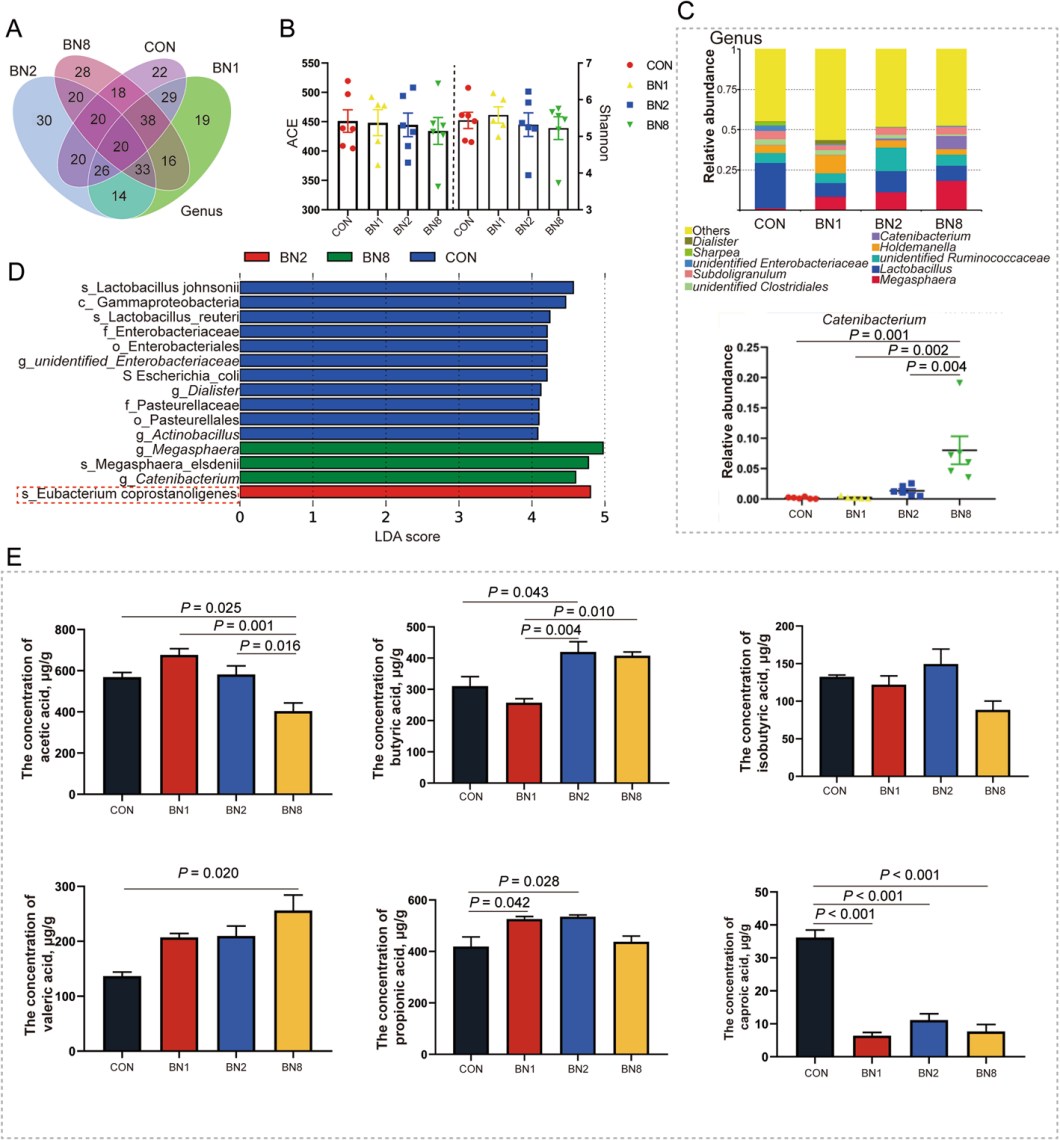
Butyrate combined with niacin regulates colonic microbial homeostasis to improve intestinal barrier function. **A** Venn diagram illustrating common and special OTUs distributed among the four groups. **B** The ACE and Shannon indices of the microbiota. **C** Composition of the colonic microbiota at the genus level. **D** LEfSe analysis (LDA score ≥ 4) was used to identify the biomarker bacterial species in different groups. **E** Quantitation of SCFAs in the colonic contents. Data are presented as mean ± SEM (*n* = 6)

The microbial community characterized by the distribution, quantity, and composition of microorganisms within the intestine constitutes part of the intestine barrier function. Therefore, we examined the composition of the colonic microbiota and its metabolite levels. A total of 483 OTUs in the colon were detected in the four groups at the phylum level (Fig. 4A). The ACE and Shannon indices of the microbiota in the BN1, BN2, and BN8 groups were not significantly different from those in the CON group (*P* > 0.05, Fig. 4B). The relative abundance of *Catenibacterium* in the BN8 group was higher than that in the CON, BN1, and BN2 groups (*P* < 0.05, Fig. 4C). The linear discriminant analysis Effect Size (LEFSe) analysis revealed that *Megasphaera*, *Megasphaera elsdenii*, and *Catenibacterium* were more abundant in the BN8 group (Fig. 4D). We observed that *Eubacterium coprostanoligenes* was more abundant in the BN2 group. Then, we further examined the levels of SCFAs. We found that the concentration of propionic acid in the colon was higher in the BN1 group than in the CON group (*P* = 0.042, Fig. 4E). The concentrations of butyric acid and propionic acid in the colon were higher in the BN2 group than in the CON group (*P* < 0.05). When the combined ratio of butyrate and niacin was increased to 100:8, the concentration of valeric acid were increased in the colon, but the concentration of acetic acid was decreased in the colon compared with that in the CON group (*P* < 0.05). Moreover, the concentration of caproic acid in the colons of the BN1, BN2, and BN8 groups was lower than that in the colons of the CON group (*P* < 0.05). These results indicate that BN2 more effectively promotes energy metabolism and barrier function in the colon of weaned piglets. Therefore, we selected BN2 for further investigation of its role in resisting ETEC infection.

### Experiment 2: growth performance and colonic barrier function of weaned piglets

**Fig. 5:**
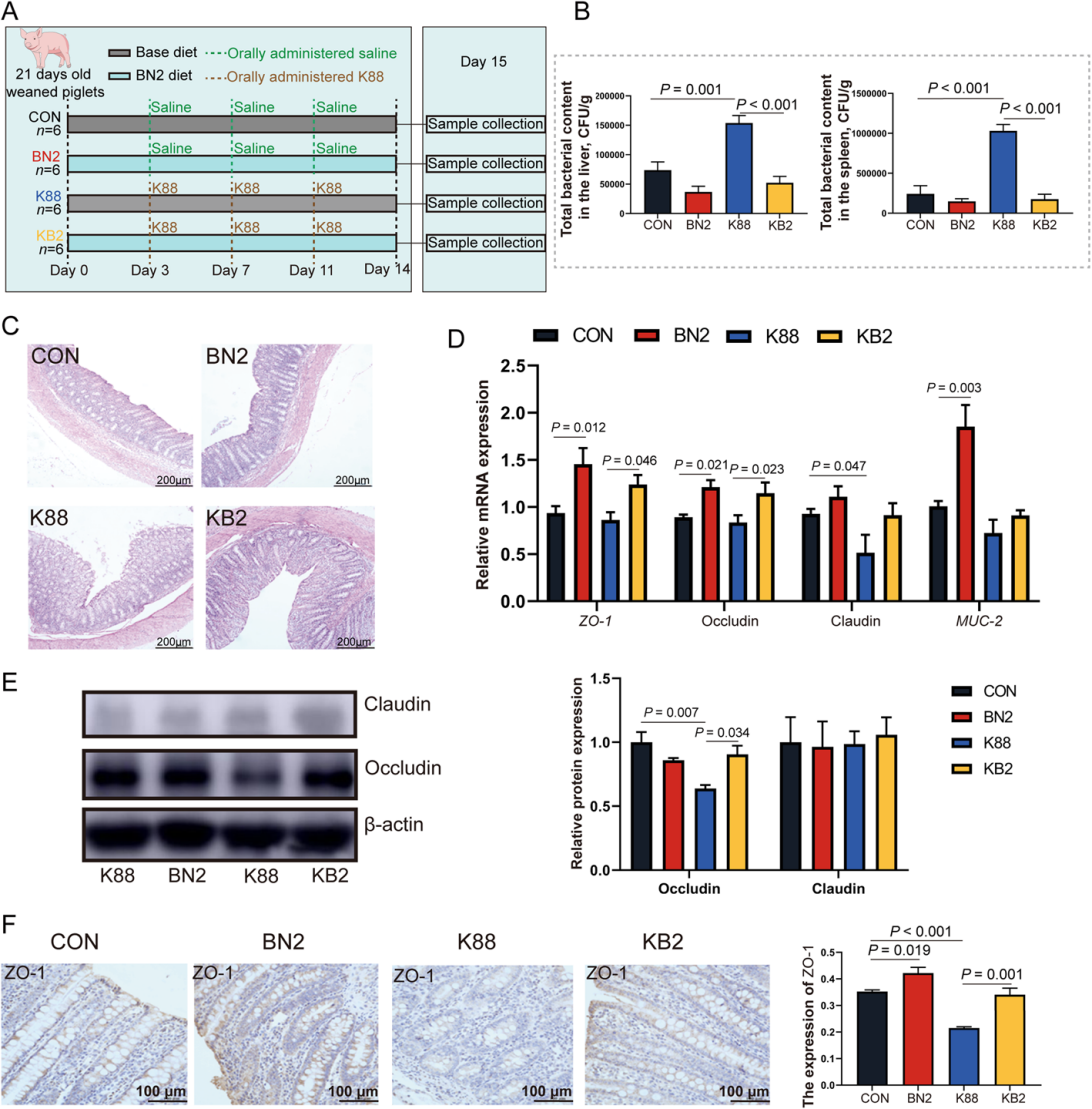
Butyrate combined with niacin alleviates ETEC-induced growth retardation and colonic barrier dysfunction in weaned piglets. **A** Experimental design for Exp. 2. **B** Total bacterial content in the liver and spleen of weaned piglets. **C** Images of HE staining in the colon (scale bar = 100 µm). **D** The mRNA expression of tight junction proteins (*ZO-1*, occludin, and claudin) and *MUC-2* in the colon. **E** Protein bands and quantitative analysis of claudin and occludin in the colon. **F** Immunohistochemistry images of ZO-1 in the colon and quantitative analysis (scale bar = 100 µm). Data are presented as mean ± SEM (*n* = 6)

To evaluate the effectiveness of butyrate combined with niacin supplementation in mitigating bacterial infections, this study established an ETEC infection model by administering ETEC K88 to weaned piglets (Fig. 5A). We observed that the final BW, ADG, ADFI, and G/F of pigs were greater in the BN2 group than in the CON group (*P* < 0.05, Table 3). The final BW, ADG, ADFI, and G/F of pigs were lower in the K88 group than in the CON group (*P* < 0.05). Moreover, the final BW, ADG, ADFI, and G/F of pigs were greater in the KB2 group than in the K88 group (*P* < 0.05). We also found that the diarrhea incidence in the K88 group was higher than that in the CON group, whereas the diarrhea incidence of pigs in the KB2 group was lower than that in the K88 group (*P* < 0.05). In the liver and spleen, the total bacterial counts in the K88 group were higher than those in the CON group (*P* < 0.05), whereas the total bacterial counts in the KB2 group were lower than those in the K88 group (*P* < 0.001, Fig. 5B). We observed that the colonic morphology was more intact in the BN2 group, whereas damage to the colonic mucosa was detected in the K88 group (Fig. 5C). In addition, the mRNA levels of *ZO-1*, occludin, and *MUC-2* in the colon were greater in the BN2 group than in the CON group (*P* < 0.05, Fig. 5D). The mRNA level of claudin was lower in the K88 group than in the CON group (*P* = 0.047), but the protein level of claudin did not change among the four groups (*P* > 0.05, Fig. 5E). Notably, the protein level of occludin in the colon was lower in the K88 group than in the CON group (*P* = 0.007). We also found that the protein level of occludin in the colon were greater in the KB2 group than in the K88 group (*P* = 0.034). Next, we validated ZO-1 protein expression using immunohistochemistry. Compared with the CON group, the protein expression of ZO-1 in the colon was lower in the K88 group (*P* < 0.001, Fig. 5F). Compared with that in the K88 group, the protein expression of ZO-1 in the colon was greater in the KB2 group (*P* = 0.001).

**Table 3:** Effects of butyrate combined with niacin on growth performance and diarrhea incidence in weaned piglets infected with ETEC

Items	CON	BN2	K88	KB2	SEM	*P*-value
Initial BW, kg	6.602	6.608	6.617	6.602	0.0575	0.999
Final BW, kg	10.35^b^	11.34^a^	8.69^c^	10.57^b^	0.217	< 0.001
ADG, g	267.4^b^	338.0^a^	148.2^c^	283.3^b^	15.54	< 0.001
ADFI, g	373.4^b^	442.0^a^	226.9^c^	385.6^b^	18.39	< 0.001
G/F	0.7166^b^	0.7649^a^	0.6535^c^	0.7347^b^	0.00516	< 0.001
Diarrhea incidence, %	22.23^b^	16.70^b^	67.86^a^	29.49^b^	4.699	< 0.001

Mean and total SEM are listed in separate columns (*n* = 6)

^a,b^Different superscript letters within a row indicate significant differences (*P* < 0.05)

*BW* Body weight, *ADG* Average daily gain, *ADFI* Average daily feed intake, *G/F* Ratio of gain to feed

### Experiment 2: colonic immune homeostasis and antimicrobial peptide expression of weaned piglets

**Fig. 6:**
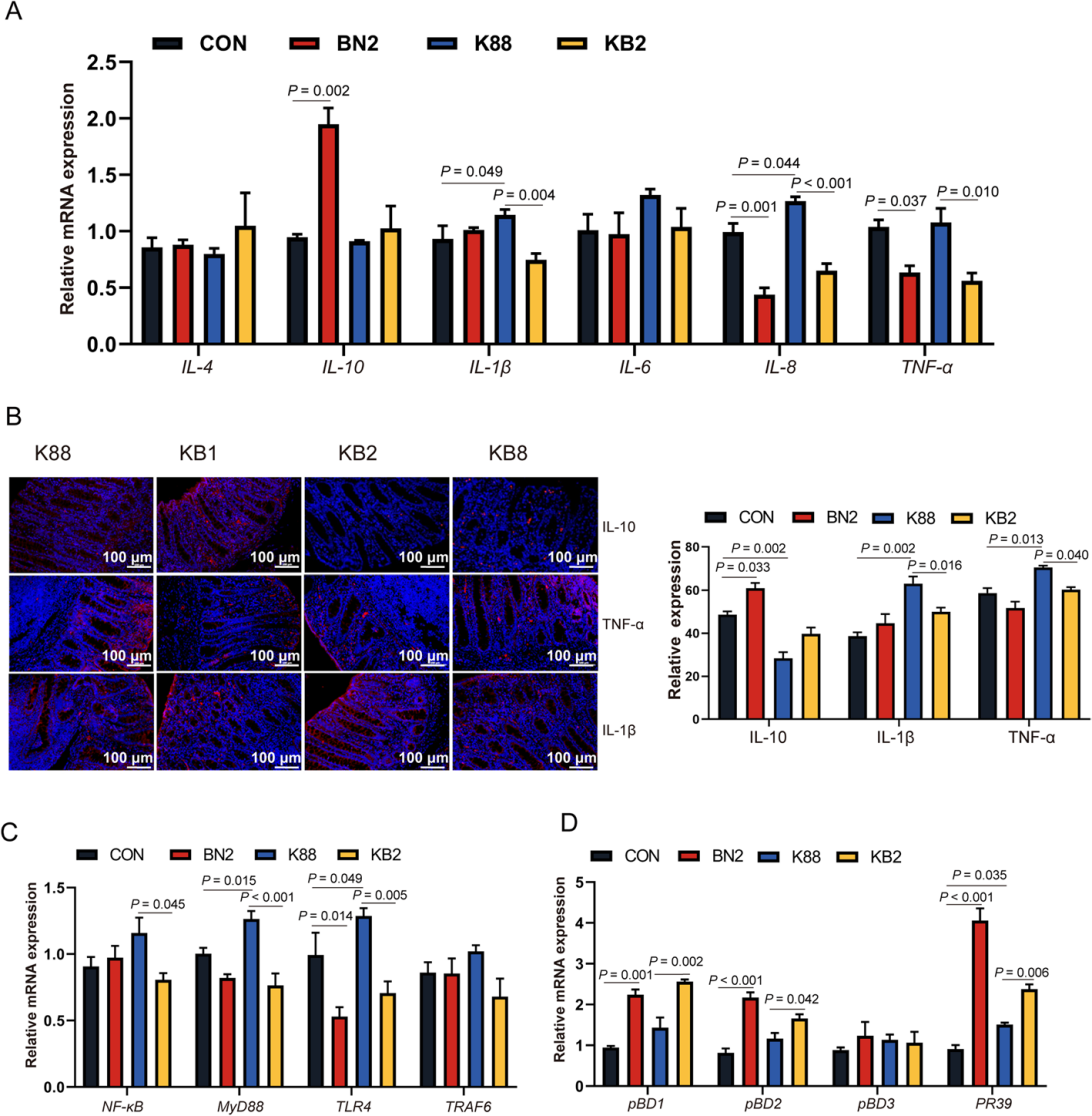
Butyrate combined with niacin enhances colonic antimicrobial peptide expression to mitigate ETEC-induced colonic barrier dysfunction. **A** The mRNA expression of inflammatory factors (*IL-4*, *IL-10*, *IL-1β*, *IL-6*, *IL-8*, and *TNF-α*) in the colon. **B** Immunofluorescence images and quantitative analysis of IL-10, IL-1β, and TNF-α in the colon. **C** The mRNA expression of immunomodulatory signaling pathway genes (*NF-κB*, *MyD88*, *TLR4*, *TRAF6*) in the colon. **D** The mRNA expression of antimicrobial peptides (*pBD1*, *pBD2*, *pBD3*, and *PR39*) in the colon. Data are presented as mean ± SEM (data from Western bolt, *n* = 3; other data, *n* = 6)

This investigation further highlighted the significant impact of butyrate combined with niacin on colonic immune barrier function induced by ETEC K88 in weaned piglets. Notably, the mRNA levels of proinflammatory cytokines (*IL-1β* and *IL-8*) in the colon were increased in the K88 group compared with the CON group** (***P* < 0.05, Fig. 6A). Compared with the CON group, the BN2 group increased the mRNA level of anti-inflammatory cytokine (*IL-10*) in the colon and decreased the mRNA level of proinflammatory cytokines (*IL-8* and *TNF-α*) in the colon (*P* < 0.05). Immunofluorescence analysis further confirmed the K88 group presented a reduction in the protein level of IL-10 and an increase in the protein levels of IL-1β and TNF-α in the colon compared with the CON group (*P* < 0.05, Fig. 6B). In contrast, the protein levels of IL-1β and TNF-α in the colon were lower in the KB2 group than in the K88 group (*P* < 0.05). In addition, the protein level of IL-10 in the colon was higher in the BN2 group than in the CON group (*P* = 0.002). Furthermore, the mRNA levels of toll-like receptor 4 (*TLR4*) and myeloid differentiation primary response 88 (*MyD88*) in the colon were higher in the K88 group compared with the CON group (*P* < 0.05, Fig. 6C). In contrast, there was a decreased in the mRNA expression of *TLR4*, nuclear factor κB (*NF-κB*), and *MyD88* in the colon in the KB2 group compared with the K88 group (*P* < 0.05). To further investigate the changes in antimicrobial peptides during ETEC-induced impairment of colonic barrier function in piglets, the mRNA expression levels of *pBD1*, *pBD2*, *pBD3*, and *PR39* were subsequently examined. The BN2 group presented increased the mRNA expression of *pBD1*, *pBD2*, and *PR39* compared with the CON group (*P* < 0.05, Fig. 6D). The mRNA expression levels of *pBD1*, *pBD2*, and *PR39* remained higher in the KB2 group than in the K88 group (*P* < 0.05).

### Experiment 1 and experiment 2: histone acetylation regulates antimicrobial peptide expression

**Fig. 7:**
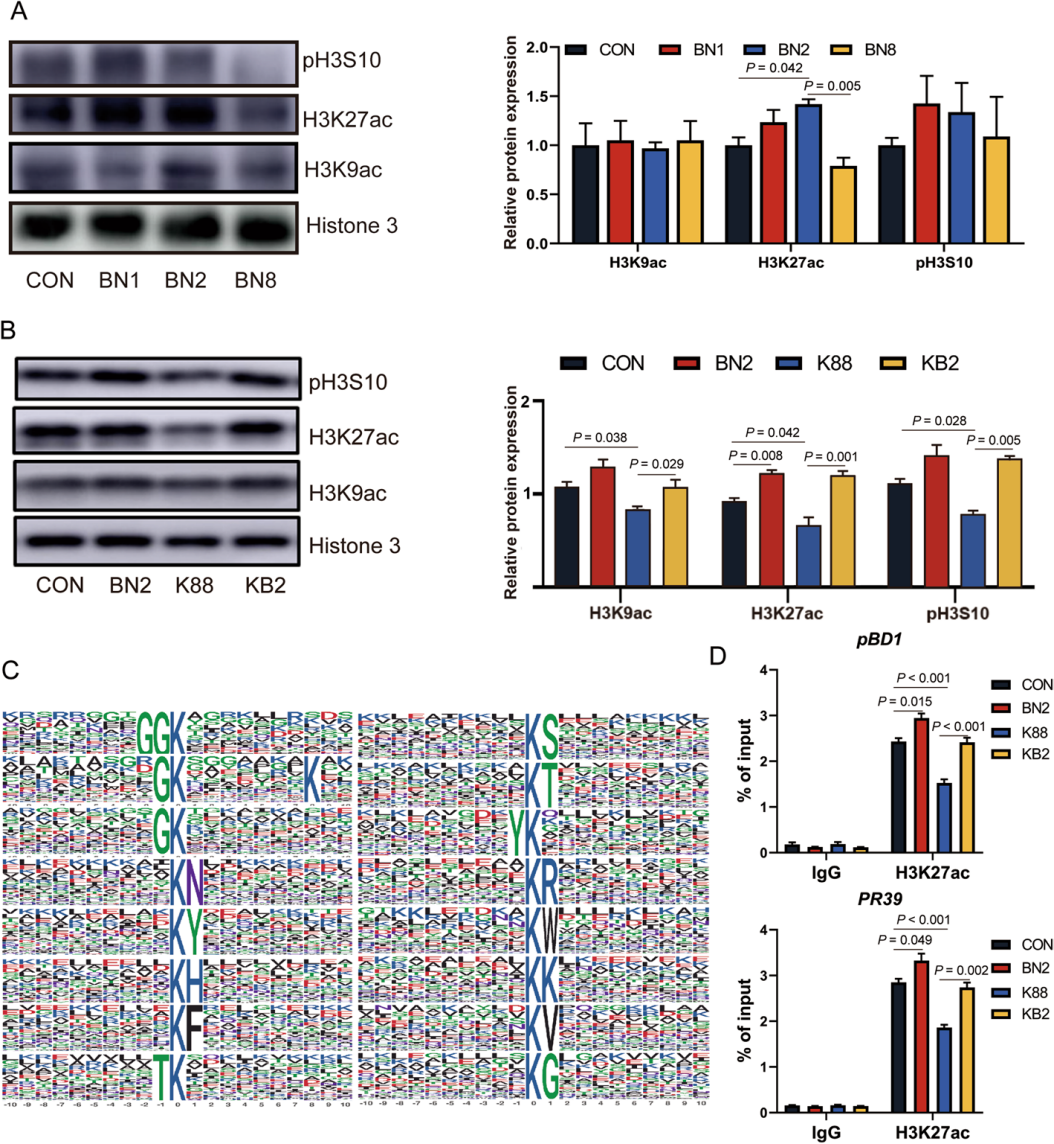
Butyrate combined with niacin enhanced colonic histone acetylation promotes antimicrobial peptide expression. Protein bands and quantitative analysis of H3K9ac, H3K27ac and pH3S10 in the colon of weaned piglets in Exp. 1 (**A**) and Exp. 2 (**B**). **C** Predicted binding map of the *pBD1* and *PR39* promoter sites. **D** Detection of H3K27ac binding to the *pBD1* and *PR39* promoters in colonic tissues from in Exp. 2 via ChIP‒qPCR. Data are presented as mean ± SEM (data from Western bolt, *n* = 3; other data, *n* = 6)

To further validate the molecular mechanism by which butyrate combined with niacin promotes the transcription of antimicrobial peptides, we examined the protein expression of H3K9ac, H3K27ac, and pH3S10 in the colon of piglets from Exp. 1 and Exp. 2 via Western blot. Notably, the protein expression of H3K27ac in the colon of the BN2 group higher than those in both CON in Exp. 1 and Exp. 2 (*P* < 0.05, Fig. 7A). In addition, the protein levels of H3K9ac, H3K27ac, and pH3S10 in the colon were lower in the K88 group than in the CON group (*P* < 0.05, Fig. 7B). However, the protein expression levels of H3K9ac, H3K27ac, and pH3S10 in the colon were higher in the KB2 group than in the K88 group (*P* < 0.05). These findings suggest that the ability of butyrate combined with niacin to promote the expression of antimicrobial peptides may be related to histone acetylation, particularly H3K27ac. We further performed motif analysis and identified amino acid sequence patterns in the region of the acetylation site (Fig. 7C). These sequences suggest that proteins containing specific amino acid residues around lysine residues in the colon are more likely to be modified by acetyl groups. To determine whether *PBD1* and *PR39* expression is involved in histone modification in the present study, we performed ChIP-qPCR to detect the *PBD1* and *PR39* promoter occupancy in the region of H3K27ac enrichment in the colon of piglets from Exp. 2. We found that the BN2 group increased the binding rate of H3K27ac with *pBD1* and *PR39* promoter regions compared with the CON group (*P* < 0.05, Fig. 7D). The binding rate of H3K27ac with *pBD1* and *PR39* promoter regions was lower in the K88 group than in the CON group (*P* < 0.05). In contrast, the binding rate of H3K27ac with *pBD1* and *PR39* promoter regions was higher in the KB2 group than in the K88 group (*P* < 0.05).

## Discussion

Early weaning can cause both nutritional and environmental stress in piglets, leading to impaired intestinal function, reduced immunity, and growth retardation [2]. Additionally, weaning stress makes piglets more susceptible to ETEC infection, exacerbating colonic dysfunction and diarrhea [3]. Butyrate serves as an energy substrate for the gut and an inhibitor of histone deacetylases, playing a crucial role in regulating the intestinal barrier function [12, 16]. Niacin is a key precursor for NAD synthesis and participates in regulating energy metabolism processes [9]. This suggests that butyrate combined with niacin may enhance intestinal energy metabolism and barrier function, thereby further alleviating ETEC-induced colonic barrier dysfunction in weaned piglets. The colon contains a rich microbial community and is the primary site where ETEC colonizes and causes diarrhea in piglets. To enhance the release of butyrate and niacin in the large intestine for metabolic regulation and restoration of the intestinal barrier function, this study employed a dual-coating technique to reduce their release rates in the stomach and small intestine of piglets. An in vitro digestion simulation study reported that less than 5% of the coated butyrate was released in the stomach, while approximately 75% was released in the small intestine [17]. This suggests that the coating technology can significantly reduce the release of coated butyrate and niacin in both the stomach and small intestine, with more than 25% of the butyrate and niacin reaching the colon. Obviously, the present study showed that among the three dietary treatments (BN1, BN2, and BN8), only the BN2 treatment increased colonic NAD synthesis and promoted colonic amino acid synthesis, glycolysis, and TCA cycle. Additionally, the BN2 treatment resisted ETEC infection by regulating the expression of tight junction proteins and cytokines. Importantly, the BN2 treatment promoted the expression of antimicrobial peptides in the colon, a finding observed in both ETEC-infected and uninfected animal models. In summary, supplementation with BN2 promoted growth performance, supported colonic health, and enhanced disease resistance in weaned piglets challenged with ETEC.

In the present study, the Exp. 1 showed that among the three dietary treatments (BN1, BN2, and BN8), only the BN2 treatment increased the ADG and G/F of weaned piglets. This highlights the advantage of a 100:2 ratio of butyrate to niacin in improving growth performance in weaned piglets. This may be related to alterations in colonic metabolism and barrier function. To investigate this, we first evaluated the effects of butyrate combined with niacin on colonic metabolism of weaned piglets. Niacin is enzymatically converted into NAD through the Preiss-Handler pathway [9]. NAD redox is essential for a variety of biochemical reactions that depend on electron exchange and is primarily a cofactor for 1) glycolysis, 2) oxidative decarboxylation of pyruvate to acetyl coenzyme A, 3) the TCA cycle, and 4) fatty acid β-oxidation [18]. In the present study, the Exp. 1 showed that the BN2 treatment increased the concentrations of NAD in the colon of weaned piglets. To further verify the influence of butyrate combined with niacin at the metabolic level in the colon of weaned piglets, we measured the colonic metabolites via metabolomics analysis. The results from Exp. 1 showed that the BN2 treatment primarily increased the contents of amino acids and vitamins in the colon, such as L-glutamine, L-leucine, D-proline, L-tyrosine, L-phenylalanine, Pro-Val, N(alpha)-acetyl-L-lysin, pantothenate, nicotinate, thiamine, and pyridoxal (vitamin B_6_). Amino acid-derived carbon readily fuels the TCA cycle and fatty acid synthesis, with amino acid oxidation accounting for 33% of mitochondrial oxidative phosphorylation [19]. Therefore, the promotion of colonic amino acid synthesis by the BN2 treatment may be associated with metabolic shifts.

In addition, the results from Exp. 1 revealed that posttranslational modification (acetylation) proteins were also enriched in the TCA cycle (IDH2, MDH1, ACLY, and SUCLG2) after the BN2 treatment. We also found that ACLY activity was increased after BN2 treatment but did not change after BN8 treatment. This may be because the enzymatic activity of ACLY fluctuates with the corresponding availability of citric acid [20]. Furthermore, the increase in HK, PK, and SDH activities confirm that the BN2 treatment promoted glycolysis and the TCA cycle. Previous study have showed that acetylation plays an important role in regulating glycolysis, the TCA cycle, and fatty acid metabolism and that the concentration of metabolic fuels (glucose, amino acids, and fatty acids) affects the acetylation status of metabolic enzymes [21]. TCA cycle bridges carbohydrate, fatty acid and amino acid metabolism [22]. This suggests that the BN2 treatment regulates colonic metabolism, potentially by promoting glucose oxidation to reduce amino acid entry into the TCA cycle for oxidative metabolism, thereby achieving amino acid conservation and synthesis. However, as the ratio of butyrate to niacin increases, the BN8 treatment exhibits an opposite effect on the regulation of colonic metabolism. Previous studies have reported that microorganisms in the intestinal lumen convert nicotinamide into niacin [23]. We hypothesize that after piglets consume excessive amounts of niacin, the microbial conversion of nicotinamide into niacin also increases. This leads to the accumulation of niacin in the intestinal lumen, triggering negative feedback regulation that reduces NAD synthesis. Overall, dietary supplementation with the BN2 diet regulated colonic metabolism of piglets by altering colonic amino acid metabolites, glycolysis and the TCA cycle. These changes may contribute to improved growth performance and enhanced resistance to ETEC infection in weaned piglets.

Impairment of intestinal morphology and dysfunction of the intestinal barrier function can lead to increase susceptibility to pathogen, which can induce the occurrence and progression of intestinal and other diseases. Butyrate and niacin have beneficial effects on providing energy to intestinal cells, thus improving the intestinal barrier and intestinal immunity. Previous studies have indicated that dietary supplementation with butyrate or niacin alone can promote intestinal barrier function and intestinal immunity [24–26]. Research has reported that niacin supplementation can reduce intestinal damage in weaned piglets by increasing *ZO-1* expression [24]. In the present study, the results from Exp. 1 showed that the colonic morphological structures of weaned piglets in all treatments were essentially the same, except in the BN8 treatment, where the submucosa of the colon was loose. The results from Exp. 1 showed that the BN2 treatment enhanced intestinal barrier function in piglets by increasing the expression of tight junction proteins, *MUC-2*, and antimicrobial peptides (*pBD1*, *pBD2*, and *PR39*) in the colon of weaned piglets. As a crucial component of the specific immune response, antimicrobial proteins serve as the first line of defense against intestinal toxins and pathogenic microorganisms [27]. These results indicate that the BN2 treatment promoted antimicrobial peptide expression to further improved colonic barrier function.

Crosstalk between nutrients and microorganisms, which are components of the intestinal barrier, is crucial. Previous studies have showed that the diversity and abundance of the intestinal microbiota are influenced by butyrate and niacin [28, 29]. In our study, the results from Exp. 1 showed that an increase in the abundance of *Eubacterium coprostanoligenes* in the colon after the BN2 treatment, whereas the relative abundance of *Catenibacterium, Megasphaera,* and *Megasphaera elsdenii* increased after the BN8 treatment. Research indicates that *Eubacterium coprostanoligenes* promotes the production of multiple SCFAs in the intestines and enhances the intestinal mucus barrier [30, 31]. Indeed, the results from Exp. 1 showed that the BN2 treatment increased the contents of butyric acid and propionic acid. *Megasphaera,* and *Megasphaera elsdenii* ferment amino acids into ammonia and branched-chain fatty acids, and causes disruption of colonic epithelial homeostasis and inflammation [32, 33]. Recent reports have indicated that *Catenibacterium* disrupts the intestinal barrier [34]. This may explain why the BN8 treatment reduced colonic acetate and propionate levels without improving colonic barrier function. These findings indicated that the BN2 treatment improved colonic barrier function in weaned piglets, which is closely associated with changes in microbial composition and metabolites.

Next, we evaluated the mitigating effect of BN2 treatment on ETEC-induced colonic barrier dysfunction in weaned piglets. ETEC colonize the intestinal epithelium through adhesins and exert their pathogenicity by releasing either heat-labile or heat-stable enterotoxins, resulting in the loss of electrolytes and water from the intestinal lumen [4]. For young animals just beginning to establish their own microbial communities, ETEC-induced diarrhea and dehydration can lead to malnutrition, impact the immune system, and may even be fatal [35]. Therefore, in the early stages of animal life, establishing stable colonic function through nutritional intervention is crucial. The results from Exp. 2 showed that K88 treatment reduced the ADG, ADFI, and G/F of weaned piglets. However, KB2 treatment had the opposite effect, reversing these reductions. These results revealed that BN2 retains potential for overcoming growth restrictions caused by ETEC infection. Previous studies have indicated that ETEC infection leads to diarrhea, disrupts the integrity of the intestinal structure, and reduces the expression of tight junction proteins such as claudin, occludin, and *ZO-1* [36–38]. In the present study, the KB2 treatment increased the expression of occludin and *ZO-1*, maintained the colonic barrier function, and reduced the transfer of bacteria to organs. ETEC-induced intestinal barrier damage disrupts the balance between proinflammatory and anti-inflammatory cytokines, leading to an inflammatory response [39]. ETEC infection increase the expression of proinflammatory factors and activate the TLR4/MyD88 pathway, exacerbating intestinal damage [40]. In this study, the results from Exp. 2 showed that the KB2 treatment inhibited the activation of TLR4/MyD88 pathway, thereby reversing the inflammatory response caused by ETEC infection. In addition, the results from Exp. 2 also indicated that KB2 treatment reversed the reduction in the expression of TJ protein and antimicrobial peptide (*pBD1*, *pBD2*, *PR39*) in the colon of weaned piglets caused by K88 treatment. This indicated that an appropriate ratio of butyrate to niacin (BN2) offers a distinct advantage in mitigating intestinal damage caused by ETEC in piglets, a finding that may also be closely linked to antimicrobial peptide expression. β-defensins inhibit the activation of the ECC-induced TLR4-NF-κB signaling pathway, thereby reducing the expression of intestinal mucosal inflammatory mediators IL-1β and TNF-α [41]. PR39 was initially identified from the gut of pigs and exhibits specific antibacterial activity against multiple Gram-negative bacteria [42]. Taken together, the KB2 treatment enhanced colonic immune responses and barrier function by increasing antimicrobial peptide expression, thereby counteracting colonic barrier dysfunction induced by ETEC infection.

**Fig. 8:**
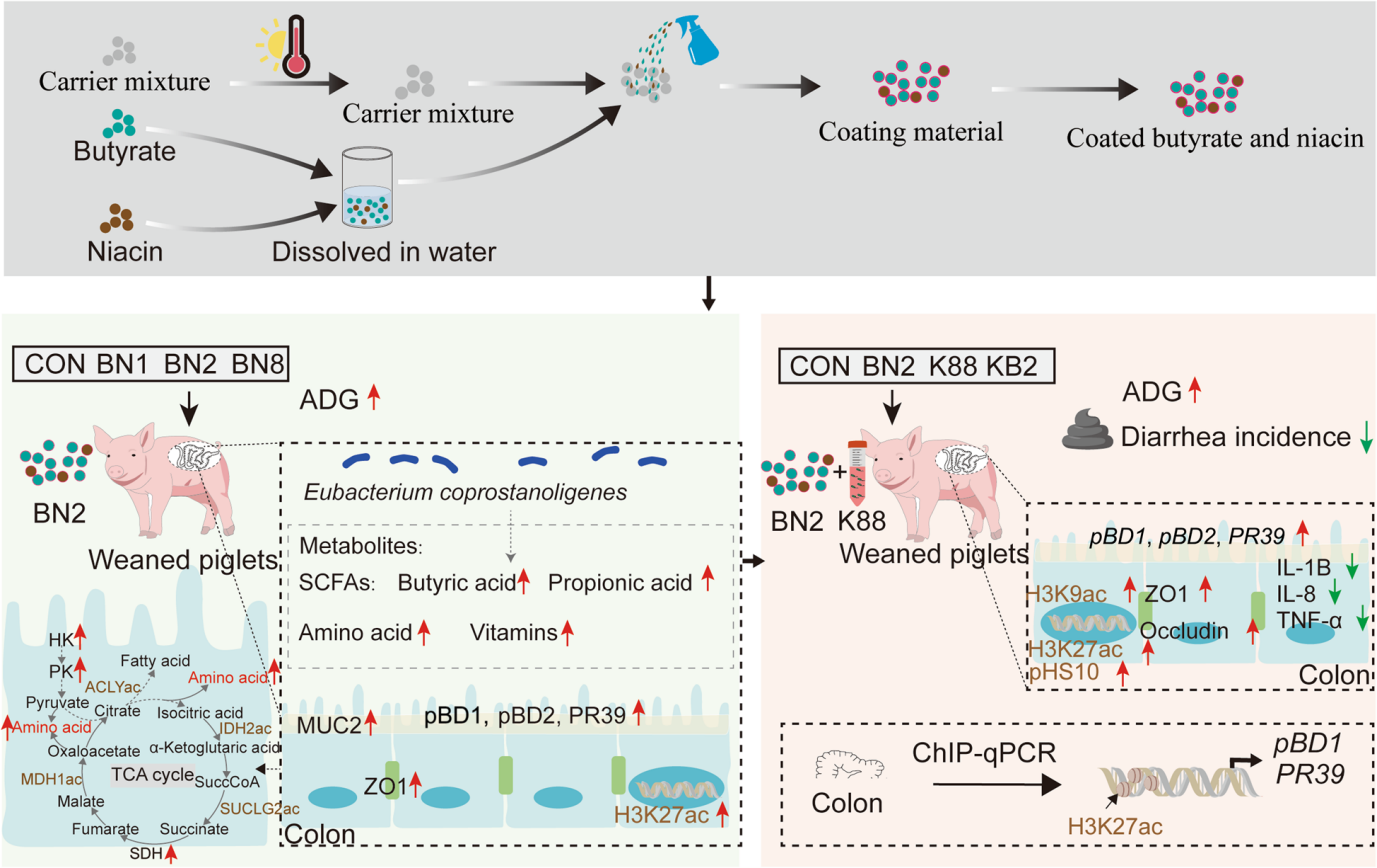
Proposed mechanisms by which butyrate combined with niacin improves clonic barrier function and energy metabolism in weaned piglets. The red arrow indicates an increase in the value of the detected parameter compared with that of CON or K88, and the green arrow indicates a decrease

Given the crucial role of antimicrobial peptides in the combined effects of butyrate combined with niacin in alleviating ETEC-induced colonic barrier dysfunction, we further investigated the mechanism by which butyrate combined with niacin regulate antimicrobial peptide expression. Histone acetylation facilitates transcription factor activation by altering chromatin structure [43]. Elevated levels of acetylated histones H3K9ac, H3K527ac, and pH3S10 in the promoter region are characteristic markers of antimicrobial peptide transcriptional activation [14]. Research has showed that butyrate enhances the level of histone H3K9ac in the promoter region of intestinal antimicrobial peptides, reinforcing disease resistance in piglets [44]. Previous studies have showed that niacin effectively relieves intestinal inflammation in weaned piglets by promoting the expression of H3K9ac, H3K27ac and pH3S10 [38]. In the present study, the results from Exp. 1 and Exp. 2 showed that the expression of H3K27ac in the colon was increased after the BN2 treatment. Meanwhile, the results from Exp. 2 showed that the K88 treatment reduced the levels of the H3K9ac, H3K27ac, and pH3S10 in the colon, but these effects were reversed by the KB2 treatment. Butyrate can alleviate intestinal barrier dysfunction by inducing the expression of antimicrobial peptides [45], and niacin can also resist intestinal damage caused by porcine deltacoronavirus infection by inducing the expression of antimicrobial peptides [46]. The results from Exp. 2 showed that BN2 treatment enhanced H3K27ac enrichment at the *pBD1* and *PR39* promoters, and KB2 treatment reversed the reduction in H3K27ac enrichment at these promoters induced by K88 treatment. These results confirm that BN2 promoted antimicrobial peptide transcription and expression by enhancing H3K27ac expression. Butyrate promotes histone acetylation by inhibiting histone deacetylases, whereas niacin activates SIRTs via NAD to increase histone deacetylation [11, 12]. An increased ratio of butyrate to nicotinate may exacerbate the antagonistic effects on histone acetylation modifications, thereby reducing both histone acetylation levels and gene expression. This explains why BN8 treatment in Exp. 1 decreased H3K27ac protein expression and antimicrobial peptide expression in the colon. In conclusion, butyrate combined with niacin promotes the repair of intestinal barrier function in weaned piglets and enhances their resistance to ETEC infection. This effect is achieved through two mechanisms simultaneously: (i) the enhancement of colonic energy metabolism via the promotion of NAD synthesis and acetylation of metabolic enzymes; and (ii) the upregulation of antimicrobial peptides expression through H3K27ac regulation (Fig. 8). Our findings highlight the indispensable role of colonic metabolism and antimicrobial peptide expression in combating ETEC infection.

## Conclusion

In our comprehensive study, we elucidated that dietary supplementation with coated butyrate and niacin (specifically the BN2 treatment) improved growth performance and colonic metabolism. In addition, the BN2 treatment enhanced the colonic barrier function to alleviate ETEC-induced colonic barrier dysfunction in weaned piglets. This enhancement is closely linked to the regulation of the antimicrobial peptide expression. Mechanistically, the BN2 treatment enhanced antimicrobial peptide transcription and expression by promoting H3K27ac expression. In summary, butyrate combined with niacin offers unique benefits in controlling foodborne pathogens, promoting sustainable livestock farming, and maintaining intestinal health. However, the association between the colonic metabolic response of piglets to nutritional regulation and the promotion of intestinal barrier function repair is very limited in this paper. Future research should focus on the mechanisms underlying colonic metabolic responses to ETEC infection, as well as on the potential for nutritional interventions to modulate colonic metabolic patterns to resist ETEC infection.

## Supplementary Information

Additional file 1: Table S1. Primer sequences for the qRT-PCR analysis. Table S2. The antibodies in this study. Table S3. Primer sequences for the ChIP-qPCR analysis.

Additional file 2: Uncropped and unprocessed images of the complete gel and blot in Figs. 3C, 5G, 7A and B.

## Data Availability

All data produced and analyzed in this research can be obtained from the corresponding author upon request.

## Abbreviations

ACLY: ATP-citrate lyase
ADFI: Average daily feed intake
ADG: Average daily gain
ETEC: Enterotoxigenic* Escherichia coli*
H3K27ac: Acetylation of histone 3 lysin 27
H3K9ac: Acetylation of histone 3 lysin 9
HK: Hexokinase
IDH2: Isocitrate dehydrogenase 2
IL-10: Interleukin 10
IL-1β: Interleukin 1 beta
MDH: Methanol dehydrogenase
MUC-2: Mucin-2
MyD88: Myeloid differentiation primary response 88
NAD: Nicotinamide adenine dinucleotide
pBD1: Porcine β defensin 1
pBD2: Porcine β defensin 2
pH3S10: Phosphorylation of histone H3 at serine 10
PK: Pyruvate kinase
PR39: Proline-arginine rich 39-amino acid peptide
SCFAs: Short-chain fatty acids
SDH: Succinate dehydrogenase
SUCLG2: Succinate-CoA ligase GDP-forming subunit β
TLR4: Toll-like receptor 4
TNF-α: Tumor necrosis factor-α
ZO-1: Zona occludin-1
