# Supplementary material for: Butyrate combined with niacin enhances intestinal barrier function repair in weaned piglets infected with ETEC by promoting colonic metabolism and antimicrobial peptide expression
Source: J Anim Sci Biotechnol. 2026 May 13;17:91. doi: 10.1186/s40104-026-01405-y (PMC13169904; doi:10.1186/s40104-026-01405-y)
Supplement: Supplementary file 1 — Additional file 1: Table S1. Primer sequences for the qRT-PCR analysis. Table S2. The antibodies in this study. Table S3. Primer sequences for the ChIP-qPCR analysis. [file 40104_2026_1405_MOESM1_ESM.docx]

Table S1. Primer sequences for the qRT-PCR analysis

| Gene | Forward sequence (5′→3′) | Reverse sequence (5′→3′) |
| --- | --- | --- |
| Claudin | ACGGCCCAGGCCATCTAC | TGCCGGGTCCGGTAGATG |
| *ZO-1* | AGCCCGAGGCGTGTTT | GGTGGGAGGATGCTGTTG |
| Occludin | GCACCCAGCAACGACAT | CATAGACAGAATCCGAATCAC |
| *MUC-2* | CTGCTCCGGGTCCTGTGGGA | CCCGCTGGCTGGTGCGATAC |
| *PBD1* | TCCTTGTATTCCTCCTCA | ACACGCCTTTATTCCTTA |
| *PBD2* | CCAGAGGTCCGACCACTACA | GGTCCCTTCAATCCTGTTGAA |
| *PBD3* | GAAGTCTACAGAAGCCAAAT | GGTTTAGGGACACCGCAC |
| *PR-39* | CAAGGCCACCTCCGTTTT | GGTAACAAATAGCACCATAA |
| *IL-10* | GCTGAAGACCCTCAGGCTGA | TTGCTCTTGTTTTCACAGGGC |
| *IL-4* | TCACCTCCCAACTGATCCCA | ATGCACGAGTTCTTTCTCGC |
| *IL-1β* | CTCCAGCCAGTCTTCATTGTTC | TGCCTGATGCTCTTGTTCCA |
| *IL-8* | TTCGATGCCAGTGCATAAATA | CTGTACAACCTTCTGCACCCA |
| *IL-6* | TGGCTACTGCCTTCCCTACC | CAGAGATTTTGCCGAGGATG |
| *TNF-α* | CACGCTCTTCTGCCTACTGC | GTCCCTCGGCTTTGACATT |
| *NF-κB* | CTCGCACAAGGAGACATGAA | ACTCAGCCGGAAGGCATTAT |
| *MyD88* | TGGTGGTGGTTGTCTCTGATGA | TGGAGAGAGGCTGAGTGCAA |
| *TLR4* | CGTGCAGGTGGTTCCTAACA | AAAGGCTCCCAGGGCTAAAC |
| *β-actin* | CACGCCATCCTGCGTCTGGA | AGCACCGTGTTGGCGTAGAG |

Table S2. The antibodies in this study

| Antibody | Source | Identifier |
| --- | --- | --- |
| Anti-Occludin antibody | Abcam | ab31721 |
| Anti-Claudin 1 antibody | Abcam | ab129119 |
| Anti-ZO-1 antibody | Thermo Fisher Scientific | #61-7300 |
| Anti-MUC2 antibody | Abcam | ab134119 |
| Anti-IL-1 beta | Abcam | ab216995 |
| Anti-IL-10 antibody | Abcam | ab34843 |
| TNF-α Antibody | Cell signaling technology | #3707 |
| Anti-Histone H3 (phospho S10) | Abcam | ab14955 |
| Anti-Histone H3 (acetyl K9) antibody | Abcam | ab4441 |
| Anti-Histone H3 (acetyl K27) antibody | Abcam | ab4729 |
| Anti-Histone H3 antibody | Abcam | ab1791 |
| Beta-Actin Antibody | Affinity | T0022 |
| HRP-conjugated Affinipure Goat Anti-Mouse IgG(H+L) | Proteintech | SA00001-1 |
| HRP-conjugated Affinipure Goat Anti-Rabbit IgG(H+L) | Proteintech | SA00001-2 |

Table S3. Primer sequences for the ChIP-qPCR analysis

| Gene | Forward sequence (5′→3′) | Reverse sequence (5′→3′) |
| --- | --- | --- |
| *PBD1* | GTTGTCCACTGCCAGTGGC | CTTGGTGACCCAGTCAAGGG |
| *PR39* | GTGTTGGTACTATCATCCCTCCAT | GGAGTGTAGTCAGCACTCTGTCC |
