# Supplementary material for: Butyrate combined with niacin enhances intestinal barrier function repair in weaned piglets infected with ETEC by promoting colonic metabolism and antimicrobial peptide expression
Source: J Anim Sci Biotechnol. 2026 May 13;17:91. doi: 10.1186/s40104-026-01405-y (PMC13169904; doi:10.1186/s40104-026-01405-y)

Uncropped and unprocessed images of the complete gel and blot in Figure 3C


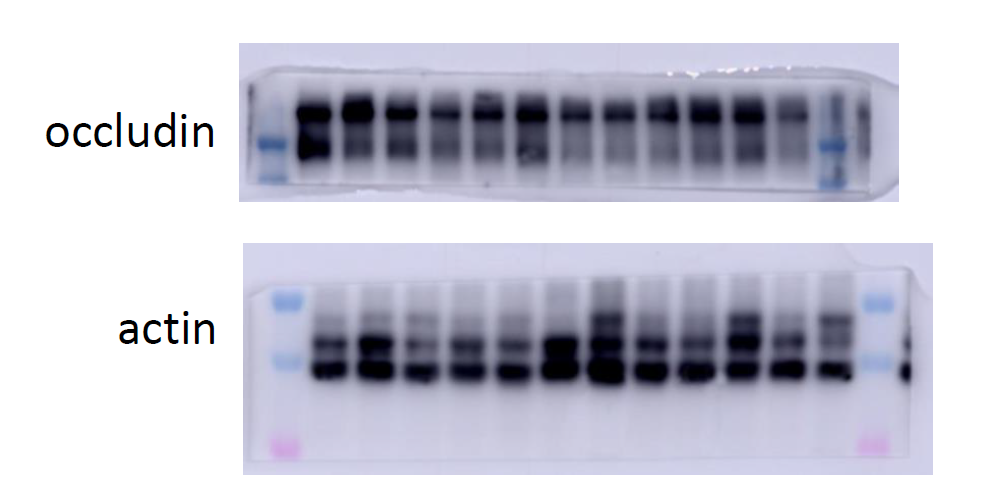


Uncropped and unprocessed images of the complete gel and blot in Figure 5G


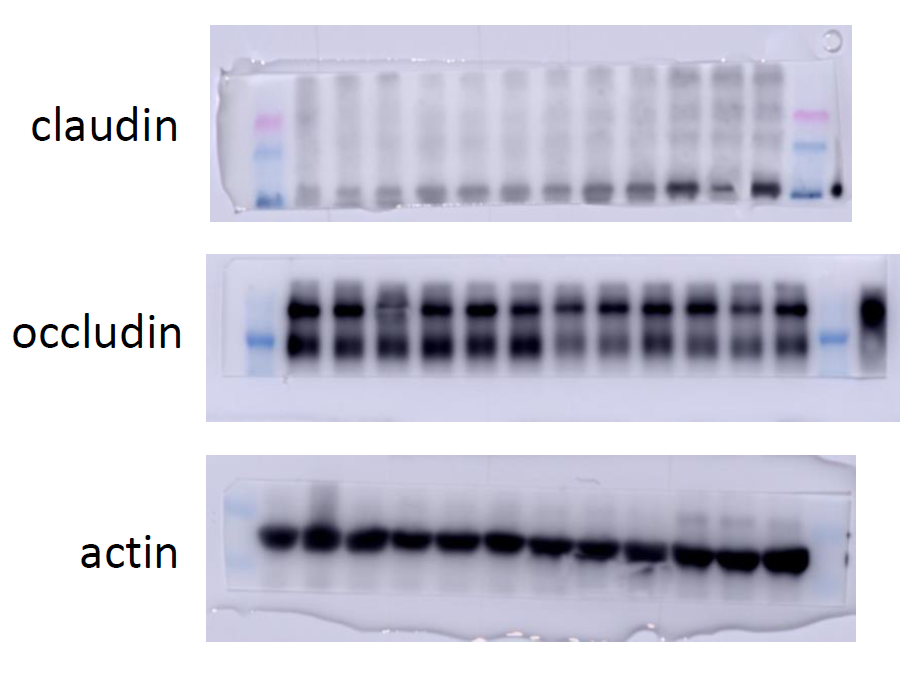


Uncropped and unprocessed images of the complete gel and blot in Figure 7A


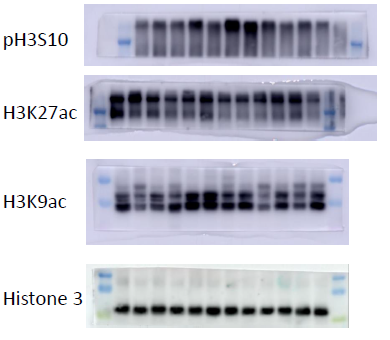

Uncropped and unprocessed images of the complete gel and blot in Figure 7B

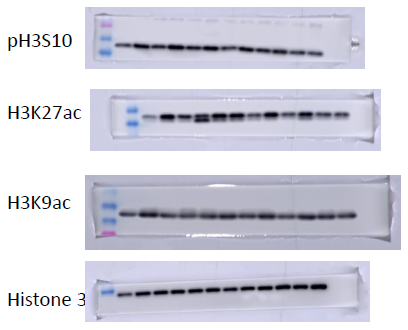

Supplement: Supplementary file 2 — Additional file 2: Uncropped and unprocessed images of the complete gel and blot in Figs. 3C, 5G, 7A and B. [file 40104_2026_1405_MOESM2_ESM.docx]
